# Insight into skywave theory and breakthrough applications in resource exploration

**DOI:** 10.1093/nsr/nwab046

**Published:** 2021-03-23

**Authors:** Qingyun Di, Changmin Fu, Guoqiang Xue, Miaoyue Wang, Zhiguo An, Ruo Wang, Zhongxing Wang, Da Lei, Xianjun Zhuo

**Affiliations:** CAS Engineering Laboratory for Deep Resources Equipment and Technology, Institute of Geology and Geophysics, Chinese Academy of Sciences, Beijing 100029, China; Key Laboratory of Shale Gas and Geoengineering, Institute of Geology and Geophysics, Chinese Academy of Sciences, Beijing 100029, China; Innovation Academy for Earth Science, Chinese Academy of Sciences, Beijing 100029, China; College of Earth and Planetary Sciences, University of Chinese Academy of Sciences, Beijing 100049, China; CAS Engineering Laboratory for Deep Resources Equipment and Technology, Institute of Geology and Geophysics, Chinese Academy of Sciences, Beijing 100029, China; Key Laboratory of Shale Gas and Geoengineering, Institute of Geology and Geophysics, Chinese Academy of Sciences, Beijing 100029, China; Innovation Academy for Earth Science, Chinese Academy of Sciences, Beijing 100029, China; College of Earth and Planetary Sciences, University of Chinese Academy of Sciences, Beijing 100049, China; Innovation Academy for Earth Science, Chinese Academy of Sciences, Beijing 100029, China; College of Earth and Planetary Sciences, University of Chinese Academy of Sciences, Beijing 100049, China; Key Laboratory of Mineral Resources, Institute of Geology and Geophysics, Chinese Academy of Sciences, Beijing 100029, China; CAS Engineering Laboratory for Deep Resources Equipment and Technology, Institute of Geology and Geophysics, Chinese Academy of Sciences, Beijing 100029, China; Key Laboratory of Shale Gas and Geoengineering, Institute of Geology and Geophysics, Chinese Academy of Sciences, Beijing 100029, China; Innovation Academy for Earth Science, Chinese Academy of Sciences, Beijing 100029, China; College of Earth and Planetary Sciences, University of Chinese Academy of Sciences, Beijing 100049, China; CAS Engineering Laboratory for Deep Resources Equipment and Technology, Institute of Geology and Geophysics, Chinese Academy of Sciences, Beijing 100029, China; Key Laboratory of Shale Gas and Geoengineering, Institute of Geology and Geophysics, Chinese Academy of Sciences, Beijing 100029, China; Innovation Academy for Earth Science, Chinese Academy of Sciences, Beijing 100029, China; College of Earth and Planetary Sciences, University of Chinese Academy of Sciences, Beijing 100049, China; CAS Engineering Laboratory for Deep Resources Equipment and Technology, Institute of Geology and Geophysics, Chinese Academy of Sciences, Beijing 100029, China; Key Laboratory of Shale Gas and Geoengineering, Institute of Geology and Geophysics, Chinese Academy of Sciences, Beijing 100029, China; Innovation Academy for Earth Science, Chinese Academy of Sciences, Beijing 100029, China; College of Earth and Planetary Sciences, University of Chinese Academy of Sciences, Beijing 100049, China; CAS Engineering Laboratory for Deep Resources Equipment and Technology, Institute of Geology and Geophysics, Chinese Academy of Sciences, Beijing 100029, China; Key Laboratory of Shale Gas and Geoengineering, Institute of Geology and Geophysics, Chinese Academy of Sciences, Beijing 100029, China; Innovation Academy for Earth Science, Chinese Academy of Sciences, Beijing 100029, China; College of Earth and Planetary Sciences, University of Chinese Academy of Sciences, Beijing 100049, China; CAS Engineering Laboratory for Deep Resources Equipment and Technology, Institute of Geology and Geophysics, Chinese Academy of Sciences, Beijing 100029, China; Key Laboratory of Shale Gas and Geoengineering, Institute of Geology and Geophysics, Chinese Academy of Sciences, Beijing 100029, China; Innovation Academy for Earth Science, Chinese Academy of Sciences, Beijing 100029, China; College of Earth and Planetary Sciences, University of Chinese Academy of Sciences, Beijing 100049, China; China Ship Research and Development Academy, Beijing 100101, China

**Keywords:** skywave theory, instrument development, electromagnetic exploration, deep resources

## Abstract

Skywave refers to the electromagnetic wave reflected or refracted from the ionosphere and propagated in the form of a guided wave between the ionosphere and the Earth's surface. Since the skywave can propagate over large distances, it has been widely used in long-distance communication. This paper explores and demonstrates the feasibility of skywave for deep resource and energy exploration at depths of up to 10 km. Theoretical and technical advancements were accomplished in furthering skywave applications. A new solution method based on Green's function has been developed to study skywave propagation in a fully coupled lithosphere-air-ionosphere full space model. For the first time, the model allows one to study skywave distribution characteristics in the lithosphere containing inhomogeneity such as ore deposits or oil and gas reservoirs. This model also lays a foundation for skywave data processing and interpretation. On a parallel line, we have developed a multi-channel, broadband, low-noise, portable data acquisition system suitable for receiving skywave signals. Using the skywave field excited by a high-power fixed source located in central China, actual field surveys have been carried out in some areas in China including the Biyang depression of Henan Province. The initial results appear encouraging—the interpreted resistivity models prove to be consistent with those of seismic exploration and known geological information, and the exploration cost is only ∼1/4 to 1/10 that of seismic surveys. These initial successful applications of the skywave theory lay a solid foundation for further verification of the new method.

## Teaser

This paper explores and demonstrates the feasibility of skywave for deep resource and energy exploration at depths of up to 10 km. Advancements were accomplished in furthering skywave applications.

## INTRODUCTION

**Figure 1.**
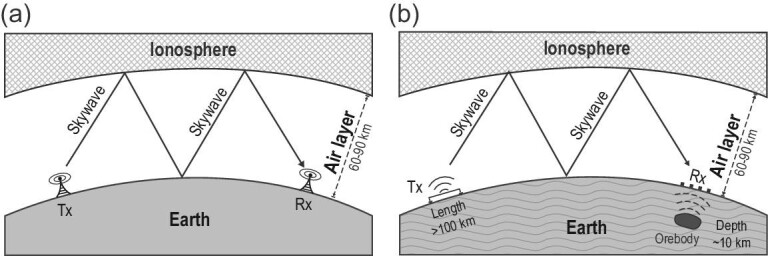
Illustrations of skywave propagation. (a) Long-distance skywave propagation for radio communication model. In the traditional radio communication model, skywave propagating in the Earth is ignored. (b) Lithosphere-air-ionosphere coupling model for skywave exploration. The distance between the receiver and the transmitter can be several thousand kilometers.

Skywave refers to electromagnetic (EM) waves propagating in the waveguide formed by the ionosphere and the Earth's lithosphere [1–7]. Due to the large contrast in the electrical property between the ionosphere and air and between the lithosphere and air, the EM waves are reflected back and forth between the ionosphere and the lithosphere as they propagate within the air waveguide. The skywave was first applied in radio communication [8–11]. Since skywave propagation is not affected by the curvature of the Earth, long-distance communication can be achieved (Fig. 1a). In traditional radio communication, high-frequency (>3 MHz) EM waves are usually used for efficient information transmission. Since the air has very high resistivity (10^13–15^ Ω m), even high-frequency EM waves can travel very far in the air, making remote communication with high-frequency radio waves possible. In the middle of the 20th century, with the widespread deployment of submarines, demand emerged for communication between land and underwater vehicles. However, because of the rapid attenuation of high-frequency EM waves in seawater, underwater vehicles could not receive signals transmitted from the land. To tackle the problem, the possibility of using low-frequency skywave for long-distance communication was explored [12–20]. For example, the United States established two super-low frequency (SLF, 30–300 Hz) transmitting stations (WTF and MTF) in Wisconsin and Michigan for communication at a distance of 4600 km and a water depth of 100 m. The former Soviet Union set up a larger SLF transmitting station (ZEVS) in the high-resistivity area of the Kola Peninsula, and successfully realized the communication between land and underwater submersibles at a distance of 8000 km and a water depth of 100 m. To further increase the depth of underwater communication, scientists from all around the world have also explored the extremely low frequency (ELF, 3–30 Hz) band [21–24].

Due to the difference in electrical properties of underground resources, the EM method has become the key technology in resource exploration [25–29]. The controlled-source audio-frequency magnetotelluric (CSAMT) method has been one of the most important methods used for resource exploration in the earth [30]. The CSAMT method has the advantages of high resolution, strong anti-interference ability and high operational efficiency, and has gradually become the mainstream method for resource exploration worldwide. However, the CSAMT method has the limitations of low transmitter power, small signal coverage and shallow sounding depth. The actual depth of investigation of the CSAMT method depends on the resistivity structure of the survey area (for the geological environment of China, the depth of investigation of the CSAMT method is usually <1.5 km). With the gradual depletion of shallow resources, demand has become increasingly high for exploration methods with larger depth of investigation [31,32].

Skywave bears great potential in resource exploration in the earth due to its large penetration depth into the lithosphere. Russian scientists have applied ZEVS signals to geophysical studies, earthquake prediction and space physics areas [33–36]. However, skywave applications for resource exploration have been scarce. The first challenge in applying skywave to resource exploration is the establishment of a proper physical model. In skywave communication, the Earth's lithosphere is assumed to be a near-perfect conductor so that it is largely excluded from the model. However, for resource exploration, this model becomes inadequate as the heterogeneities in the lithosphere now become the main target of interest. Thus, a fully coupled lithosphere, air and ionosphere model must be used (Fig. 1b). The second challenge is the lack of proper data acquisition equipment. The required equipment must be able to acquire multi-channel and wide-band data, and must have extremely low noise level. Meanwhile, the equipment must be sufficiently cost effective for large area exploration. However, no suitable instrument was available on the market to meet both the technical challenges and the economic requirements. Therefore, new equipment had to be developed. Moreover, literature on the data processing of skywave signals was rare. In this paper, we report some of the breakthroughs we have made in recent years in skywave theory and equipment development for resource exploration. We also present a field example to illustrate the application of skywave theory to oil and gas exploration.

## THEORY AND EQUIPMENT

### Solution method

To study skywave propagation in the lithosphere, a fully coupled lithosphere-air-ionosphere model is first established. This model is different from the traditional skywave model for communication in that the communication model excludes the lithosphere completely, whereas the fully coupled model enables the study of the lithosphere, including any inhomogeneity within. Also, the fully coupled model allows for the study of skywave propagation and attenuation over large distances. The model lays a foundation for skywave survey design and data interpretation.

For skywave propagation over a large distance, a spherical Earth model should be used to accurately account for the Earth’s curvature. However, for propagation distances on the order of a few hundred kilometers, a planar model can be adequate. We derive the planar full-model skywave theory by expanding the half-space CSAMT model to include ionosphere and lithosphere. The solution is achieved by the integral equation method based on the Green's function, and derived using the R-function and layer-matrix method [37,38].

### Electromagnetic field characteristics

**Figure 2.**
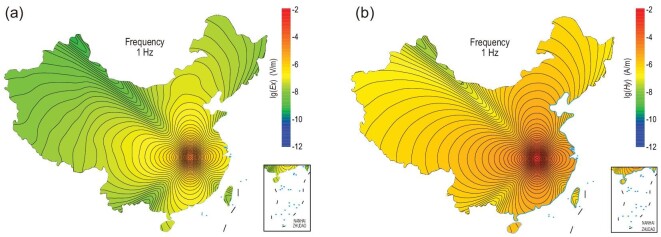
Distribution of EM fields generated by fixed sources under the national electrical structure model. The transmitting station is located in central China. (a) Electric field. (b) Magnetic field. Review drawing number: GS(2021)2093.

By using the R-function and layer-matrix method, we calculate the Green's function for the horizontal lithosphere-air-ionosphere model over the territory of China. The Green's function is used as the kernel of the integral equation method for three-dimensional (3D) modeling [39]. Figure 2 shows the model result for the electric and magnetic field distributed over the entire land area of China (see Supplementary Data for how the 3D geoelectrical model is built). For the particular transmission antenna layout, both *Ex* and *Hy* fields are divided into four quadrants (north, east, south and west). Along the NE-SW and NW-SE directions, the field strengths are locally diminished. These aerial field variations should be taken into account when doing the survey design and data interpretation.

### Equipment

**Figure 3.**
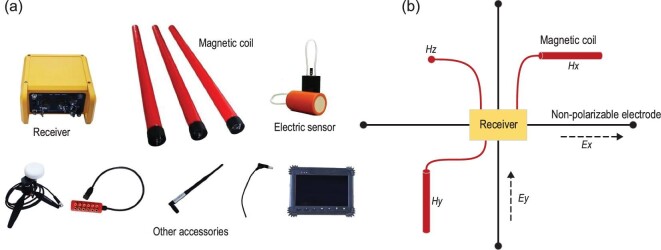
New skywave signal acquisition systems and survey arrangement in the field. (a) EM receivers, magnetic sensor and electric sensor, and accessories. (b) Survey arrangement in the field for one sounding station. For scalar observation, only *Ex* and *Hy* are observed. The non-polarizable electrodes are buried in the earth to measure electric field. The magnetic sensor is placed horizontally or vertically on the ground, and the horizontal *Hx* or *Hy* and vertical *Hz* are observed. This set-up is for tensor observation when there are two antennas.

For traditional CSAMT or magnetotelluric (MT) applications, different kinds of receivers are available on the market for EM exploration [40]. However, none of the receivers were designed for acquiring skywave signals. Their bandwidths and noise rejection capabilities appear inadequate for skywave applications. In this regard, we have developed a new receiver system for our needs. The specifications of the new broadband and low-noise system are listed in Supplementary Tables 1 and 2. Figure 3 shows the main components of the system. Field experiments proved that the newly developed acquisition system was able to acquire skywave fields for resource exploration at a depth of up to 10 km. It is worth mentioning that the instrument may also be used for traditional CSAMT and MT surveys.

## VALIDATION OF THE MEASUREMENTS

Prior to any field survey, we conducted experimental field studies to validate the skywave measurement against the well-known CSAMT and MT methods. This would establish a solid basis for performing massive skywave surveys. It is helpful to first understand how the different methods respond to a layered Earth model. For survey parameters similar to those used in this study, Supplementary Fig. 3 shows that, over all frequency ranges of interest, both the skywave and the MT responses are characterized by plan wave propagation, whereas the CSAMT method demonstrates the near-field feature at the lower frequencies due to the short transmitter-receiver spacings. This result suggests that the skywave data in the modeled transmitter-receiver distance range can be interpreted as the MT data.

Next, the skywave method was validated against the MT method over an area with low levels of EM noise. As Supplementary Fig. 4 shows, over the overlapped frequency band, the skywave sounding curves compare well in general with the MT response curves, with the skywave response being more stable than the MT response. The skywave method was further compared to both the CSAMT and MT methods in a highly noisy area. As Supplementary Figs 5 and 6 illustrate, the skywave data show good consistency over the entire frequency range, whereas the CSAMT or MT data demonstrate uninterpretable variations or outliers due to environmental noise.

## CASE STUDIES

Two skywave surveys were carried out in China. The first study area was in Chongqing, southwest China, ∼700 km away from the transmitter. The second test area was in Henan Province, central China, ∼300 km away from the transmitter. In both cases, a total of 30 sets of receiver systems were deployed.

In the Mingyuexia oil and gas area, the skywave exploration result was compared with seismic images. The skywave method was able to outline the largest regional fault, from a resistivity anomaly, as explained by the seismic reflection image. Both electrical and seismic reflection images show the typical chevron anticlines of the Huaying Mountain fold-and-thrust belt thin-skinned structures. The interested reader should refer to [41] for more details. This paper shall focus on the Biyang depression case study.

**Figure 4.**
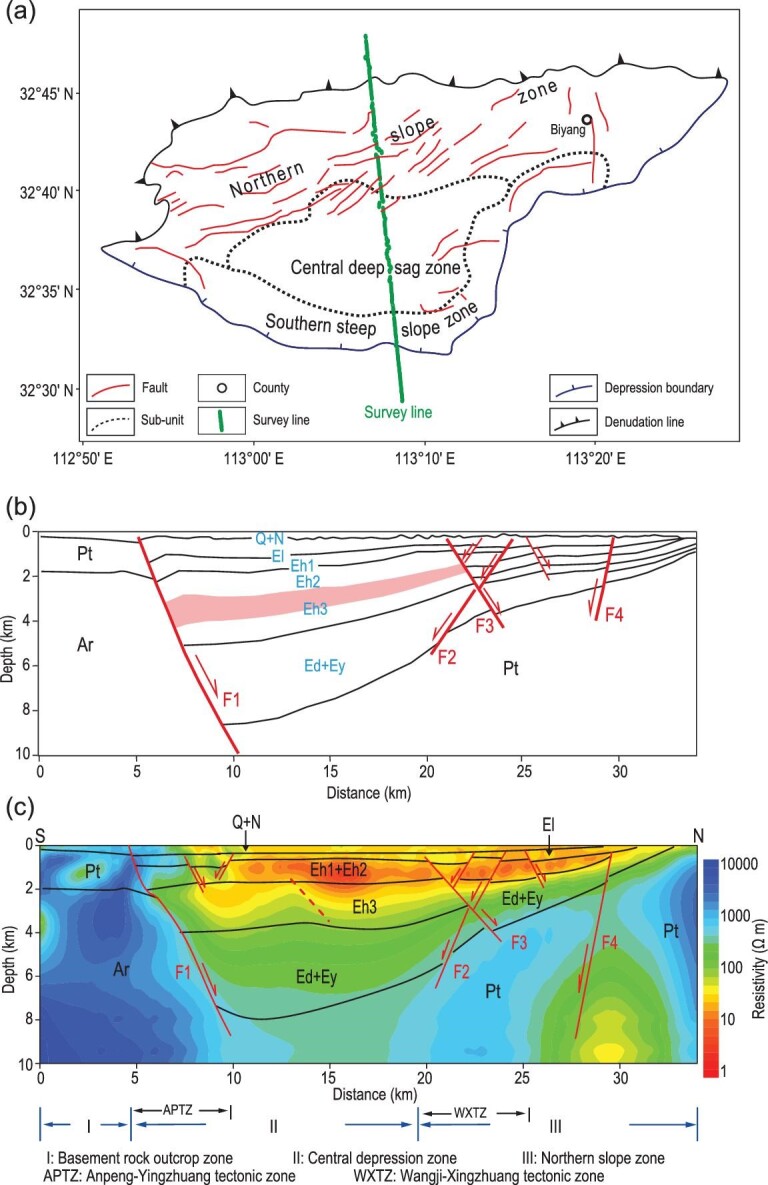
Skywave survey line in the test area of Henan Province and the inversion model. (a) Geological map of Biyang depression and the survey line. (b) Subsurface structure obtained from seismic exploration. (c) Subsurface resistivity model obtained from the skywave data. F1–4: fault. Q + N: Quaternary and Neogene deposition. Ey: Yuhuangding Formation, Paleogene. Ed: Dacangfang Formation, Paleogene. Eh: Hetaoyuan Formation, Paleogene. El: Liaozhuang Formation, Paleogene. Pt: Proterozoic.

The study area is part of the Biyang depression in the Henan Province. Figure 4a and b show the geological structure of this area as determined by previous studies. The Biyang depression is a fan-shaped depression with a southeast–northwest orientation. The major basal structures are normal faults, named F1 through F4. The primary basin fault F1 spans 5 km horizontally and is located at the southern extent of the survey line. Part of the Wangji-Xingzhuang tectonic zone (WXTZ) is generally adjacent to the northern gentle slope zone (located at distances of 25 km to 33 km in Fig. 4b) along the boundary of Fault F3 (marked in Fig. 4b). Most of the hydrocarbons discovered in the Biyang depression are distributed in the layer Eh3 (marked by the pink color in Fig. 4b).

In this survey, a transmitter in central China was used. The transmitter consists of a north–south oriented antenna that is 60 km long and located on the surface with both ends grounded in the earth. A sinusoidal current was injected into the earth through the grounded wire, and the oscillating current created EM waves that propagated outwards. During the survey, 24 frequencies were transmitted in the range of 0.1 Hz to 256 Hz (see Supplementary Table 3).

The length of the sounding profile was ∼34 km, with an azimuth of 174°, and the spacing between skywave receivers was 80 m (Fig. 4a). The signal was collected in a scalar CSAMT format in this study, where the electric field *Ex* and the magnetic field *Hy* were recorded. The data processing was also similar to that in CSAMT [42]. The apparent resistivities were used to perform a laterally constrained inversion [43,44].

The inversion model obtained from the skywave data (Fig. 4c) shows good agreement with the basin geometry. The resistivity model suggests that the study area can be divided into three parts from south to north: an outcrop zone in the south of the basement rock (I), a depression zone in the middle (II) and a gently sloping zone in the north (III). In addition, there are two areas with complex resistivity structures along the survey line: the Anpeng-Yinzhuang tectonic zone (APTZ) and the WXTZ. Combined with the stratigraphy of the study area, a comprehensive interpretation is provided in Fig. 4c.

A comparison of Fig. 4b with Fig. 4c demonstrates that the resistivity structures determined by inversion of the skywave data are consistent with the known fault structures. In particular, the hydrocarbon layer can be distinguished by the resistivity values in the range of 1–30 Ω m, thereby providing important guidance for subsequent drilling. The resistivity model also shows a deep low-resistivity zone toward the northern end of the profile that was not present in the geological mapping.

## DISCUSSION AND CONCLUSION

To apply the extremely low-frequency skywave to the exploration of deep Earth resources, the theory on the skywave wave propagation has been extended to a fully coupled lithosphere-air-ionosphere model. The R-function and layer-matrix method are used to solve the problem of skywave based on the 3D integral equation method. The inclusion of displacement currents in the model allows the accurate study of skywave propagation within the lithosphere-ionosphere waveguide on a global scale. For practical application of the skywave theory, we have developed a new multi-channel, broadband, low-noise and portable data acquisition system suitable for field surveys. Field studies have been successfully carried out in the Mingyuexia oil and gas area of Chongqing and the Biyang depression of Henan Province, and the resistivity models were derived from the skywave signal for depths up to 10 km. The obtained lithospheric models are consistent with those of seismic exploration and known geology, which validates the skywave theory and technique developed in this paper.

The skywave method has a few important advantages over the MT and CSAMT methods. By using a much more powerful transmitter than in the CSAMT method, skywaves can be detected at a distance of thousands of kilometers from the transmitter. These waves may be processed as plane waves, as in the MT method, greatly simplifying the data interpretation for the structure of the Earth's crust. Because the skywave method uses human-made sources, the signal-to-noise ratios are more controllable than for the MT method and the data quality is generally better.

Our theoretical research and field case studies show that extremely low-frequency skywave exploration may become one of the practical methods for resource and energy exploration within the upper 10 km of the crust. The cost of this method is significantly less than the seismic exploration method. In addition, the skywave method has the potential of discriminating oil or gas from water based on the resistivity contrasts of the fluids. When combined with seismic methods, the skywave method can be effectively applied in exploration of deep oil and gas.

The field examples show that the skywave signal can be stronger or even much stronger than background noises. Given the broad signal coverage, a large area (e.g. the whole of China) can be surveyed simultaneously as long as a sufficient number of survey stations cover the survey area. This can greatly reduce survey costs. Offshore application can be another area worth exploring. Extremely low-frequency skywave may supplement or supersede the traditional marine EM methods for offshore exploration.

As a resource exploration method, the extremely low-frequency skywave method warrants further study in some aspects. For example, few studies are available to address the response characteristics of the skywave in the near-field, far-field and waveguide areas. The applicability and limitations of the method for mineral exploration in these different areas call for more theoretical and field studies. Another area of study is the intermediate zone effect that describes the influence of lithospheric heterogeneities between the transmitter stations and survey areas. In this study, we have assumed that ground waves are highly attenuative and can be ignored in the acquired skywave data. Nevertheless, a detailed study of the intermediate zone effect may yield new insights into the skywave method for mineral exploration. Also, for use in areas very far from the source, it is necessary to develop the theory of a spherical layered model.

## METHODS

### R-function method

For the R-function method, the upper half-space includes two layers: air and the ionosphere. The lower half-space is an *N*-layer lithosphere, and the *N*-th layer extends downward infinitely. The integral solution of the model is derived, and the numerical solution of the lithosphere-air-ionosphere model is obtained by using the Fast Hankel transform.

The full space model consists of *N *+ 2 layers, including the ionosphere, air and *N* lithosphere layers in the lower half-space.

The field of the *p*-th layer is ${X_P} = {c_p}{e^{{u_p}{{z}}}} + {d_p}{e^{ - {u_p}z}}$, ${V_P} = c_p^*{e^{{u_p}z}} + d_p^*{e^{ - {u_o}z}}$, where $p = - 1,0,1,2 \ldots ,N$. When $p = - 1$, ${d_p} = 0$, $d_p^* = 0$, there are only upward waves. When $p = N$, ${c_N} = 0$, $c_N^* = 0$, there is only a downward wave. When the air layer contains the source, ${X_0} = {c_0}{e^{{u_0}{{z}}}} + {d_0}{e^{ - {u_0}z}} + {X_s}$, where ${X_s} = \frac{\lambda }{{{u_0}}}{e^{ - {u_0}| {{{z}} + {h_0}} |}}$ is known. The parameters ${c_p}$, ${d_p}$, $c_p^*$, $d_p^*$ that need to be solved are unknown, where $p = - 1,0,1,2, \ldots N$. After getting these parameters, we can calculate the EM field. In the solution, the R-function is defined in the lower half-space, ${R_P}( {{z}} ) = \frac{{{d_p}{e^{ - {u_p}z}} + {c_p}{e^{{u_p}z}}}}{{{d_p}{e^{ - {u_p}z}} - {c_p}{e^{{u_p}z}}}}$, $R_p^*( {{z}} ) = \frac{{d_p^*{e^{ - {u_p}z}} + c_p^*{e^{{u_p}z}}}}{{d_p^*{e^{ - {u_p}z}} - c_p^*{e^{{u_p}z}}}}$. The function at the interface of each layer can be obtained by recursions. It can further be proved that

(A1) \begin{eqnarray*} \frac{{{X_1}( 0)}}{{{{X^{\prime}}\!_1}( 0 )}} &=& - \frac{{{R_1}( 0)}}{{{u_1}}} \nonumber\\ &=& - \frac{1}{{{u_1}}}\textrm{cth}\!\left[ {u_1}{z_1} + \textrm{arcth}\frac{{{u_1}}}{{{u_2}}}\textrm{cth}\right.\nonumber\\ &&\!\!\left.\left[ {{u_2}{z_2} + \cdots + \textrm{arcth}\frac{{{u_{N - 1}}}}{{{u_{N}}}}} \right] \right],\quad\,\,\quad \end{eqnarray*}

(A2) \begin{eqnarray*} \frac{{{V_1}( 0 )}}{{{{V^{\prime}_1}}( 0 )}} &=& - \frac{{R_1^*( 0 )}}{{{{u_1}}}} \nonumber\\ &=& - \frac{1}{{{u_1}}} \textrm{cth}\left[ {u_1}{z_1} + \textrm{arcth}\frac{{{u_1{\rho_1}}}}{{{u_2{\rho_2}}}}\textrm{cth}\right.\nonumber\\ &&\left.\left[ {{u_2}{z_2} + \cdots + \textrm{arcth}\frac{{{u_{N - 1}}}}{{{u_{N}}}}\frac{{{\rho _{N - 1}}}}{{{\rho _N}}}} \right] \right].\nonumber\\\end{eqnarray*}

Since ${R_1}( 0 )$ and $R_1^*( 0 )$ are known, the ratio of the field in the first layer of solid rock ${X_1}( 0 )$, its vertical derivative${X^{\prime}_1}( 0 )$, ${V_1}( 0 )$, and its vertical derivative$V_1^{\prime}( 0 )$ are determined.

The unknown parameters in the air layer ${d_0}$, ${c_0}$, $d_0^*$, $c_0^*$ can be obtained from the four boundary conditions at the interface between the ionosphere and the air layer.

Then, we can apply the boundary conditions and the left equations in A1 and A2 for calculating the unknown parameters. The EM field components at the Earth surface can be written as

(A3) \begin{eqnarray*} {E_x} &=& {\rm{i}}\omega \mu \frac{{{P_E}}}{{4{\rm{\pi }}}}\int_{0}^{\infty }{{F \cdot }}\,{{\rm{J}}_0}(\lambda r){\rm{d}}\lambda \nonumber\\ && +\, \frac{{{\rm{i}}\omega \mu }}{{k_1^2}}\frac{{{P_E}}}{{4{\rm{\pi }}}}{{\cos }^2}\theta \int_{0}^{\infty }{{\left( {F\!F - \frac{{k_1^2}}{{{\lambda ^2}}}F} \right)\, \cdot\, }}{\lambda ^2} \nonumber\\ &&\cdot\, {{\rm{J}}_0}\,(\lambda r){\rm{d}}\lambda + \frac{{{\rm{i}}\omega \mu }}{{k_1^2}}\frac{{{P_E}}}{{4{\rm{\pi }}}}\frac{1}{r}\left( {1 - 2{{\cos }^2}\theta } \right)\nonumber\\ &&\times\int_{0}^{\infty }{{\left( {\textit{FF} - \frac{{k_1^2}}{{{\lambda ^2}}}F} \right) \,\cdot\, }}\lambda \cdot {{\rm{J}}_1}(\lambda r){\rm{d}}\lambda, \nonumber\\ [5pt] {H_y} &=& \frac{{{P_E}}}{{4{\rm{\pi }}}}\int_{0}^{\infty }{{ - \frac{{{u_1}}}{{{R_1}(0)}}F \cdot }}\,{{\rm{J}}_0}(\lambda r){\rm{d}}\lambda \nonumber\\ && +\, \frac{{{P_E}}}{{4{\rm{\pi }}}}\frac{1}{r}\left( {1 - 2{{\cos }^2}\theta } \right)\nonumber\\ &&\times\int_{0}^{\infty }\left( { - \frac{{R_1^*( 0 )}}{{{u_1}}}\textit{FF} + \frac{{{u_1}}}{{{R_1}(0)}}\frac{1}{{{\lambda ^2}}}F} \right)\lambda \nonumber\\ &&\cdot\, {{\rm{J}}_1}\,(\lambda r){\rm{d}}\lambda + \frac{{{P_E}}}{{4{\rm{\pi }}}}{{\cos }^2}\theta\nonumber\\ &&\times \int_{0}^{\infty }{{\left( { - \frac{{R_1^*( 0 )}}{{{u_1}}}\textit{FF} + \frac{{{u_1}}}{{{R_1}(0)}}\frac{1}{{{\lambda ^2}}}F} \right){\lambda ^2} }}\nonumber\\ &&\cdot\, {{\rm{J}}_0}\,(\lambda r){\rm{d}}\lambda,\end{eqnarray*}

where

(A4) \begin{eqnarray*} &&F = \frac{\lambda }{{{u_0}}}{{\rm{e}}^{ - {u_0}{h_0}}} + {e_0}\nonumber\\ &&\quad + \frac{{\left( {\frac{{\lambda {{\rm{e}}^{ - {u_0}{h_0}}}}}{{{u_0}}} + {e_0}} \right)\left( { - \frac{{{u_1}}}{{{R_1}\left( 0 \right)}}} \right) + \lambda {{\rm{e}}^{ - {u_0}{h_0}}} - {u_0}{e_0}}}{{{u_0}\frac{{{c_{oc}} - {c_{od}}}}{{{c_{oc}} + {c_{od}}}} + \frac{{{u_1}}}{{{R_1}\left( 0 \right)}}}},\qquad\qquad\nonumber\\ &&\textit{FF} = \frac{{\frac{{{{\rm{e}}^{ - {u_0}{h_0}}}}}{\lambda }\left(1\! +\! \frac{{{c_{oc}} - {c_{od}}}}{{{c_{oc}}\! +\! {c_{od}}}}\right)\! +\! {e_0}\!\left( {1 \!-\! \frac{{c_{0c}^* - c_{0d}^*}}{{c_{0c}^* + c_{0d}^*}}} \right)\frac{{c_{0c}^* + c_{0d}^*}}{{c_{0c}^* - c_{0d}^*}}}}{{\frac{{R_1^*(0)}}{{{u_1}}}\! +\! \frac{{k_0^2}}{{{u_0}k_1^2}}\frac{{c_{0c}^* + c_{0d}^*}}{{c_{0c}^* - c_{0d}^*}}}},\nonumber\\ &&{c_{oc}} = \frac{1}{2}\left( {1 + \frac{{{u_{ - 1}}}}{{{u_0}}}} \right){{\rm{e}}^{\left( {{u_{ - 1}} - {u_0}} \right){z_{ - 1}}}},\nonumber\\ &&c_{oc}^* = \frac{1}{2}\left( {1 + \frac{{{u_{ - 1}}}}{{{u_0}}}\frac{{k_0^2}}{{k_{ - 1}^2}}} \right){{\rm{e}}^{\left( {{u_{ - 1}} - {u_0}} \right){z_{ - 1}}}},\nonumber\\ &&{c_{od}} = \frac{1}{2}\left( {1 - \frac{{{u_{ - 1}}}}{{{u_0}}}} \right){{\rm{e}}^{\left( {{u_{ - 1}} + {u_0}} \right){z_{ - 1}}}},\nonumber\\ &&c_{od}^* = \frac{1}{2}\left( {1 - \frac{{{u_{ - 1}}}}{{{u_0}}}\frac{{k_0^2}}{{k_{ - 1}^2}}} \right){{\rm{e}}^{\left( {{u_{ - 1}} + {u_0}} \right){z_{ - 1}}}}.{\rm (A4)}\nonumber \end{eqnarray*}

### Layer-matrix method

For the layer-matrix method, the air layer and ionosphere in the upper half-space can have arbitrary layers, and the lithosphere layer in the lower half-space can also have arbitrary layers. The full-space Green's function in the wave number domain is derived by using the layer-matrix method, while the numerical solution of the Green's function in the frequency domain can be obtained by Fourier integration.

In the layer-matrix method, the air layer is set as the 0-th layer, the ionosphere is composed of *M*-layers and the lithosphere layer is composed of *N*-layers. The depth of the first layer in the upper half-space is ${z_{ - p}}$, where $p = 1,2, \ldots ,M$, the depth of the lower half-space is ${z_p}$, where$p = 1,2, \ldots ,N$. It can be deduced that

(A5) \begin{eqnarray*} \left[ {\begin{array}{@{}*{1}{c}@{}} {{u_x}( {{z_1}} )}\\ {{{u^{\prime}}\!_x}( {{z_1}} )}\\ {{V_z}( {{z_1}} )}\\ {{{V^{\prime}}\!_z}( {{z_1}} )} \end{array}} \right] &=& a_{{R_1}}^{ - 1}a_{{R_2}}^{ - 1} \cdots a_{{R_p}}^{ - 1} \cdots a_{{R_{N + 1}}}^{ - 1}\nonumber\\ &&\times\left[ {\begin{array}{@{}*{1}{c}@{}} {{u_x}( {{z_N}} )}\\ {u_x^{\prime}( {{z_N}} )}\\ {{V_z}( {{z_N}} )}\\ {{{V^{\prime}}\!_z}( {{z_N}} )} \end{array}} \right],\end{eqnarray*}

(A6) \begin{eqnarray*} \left[ {\begin{array}{@{}*{1}{c}@{}} {{u_x}( {{z_{ - 1}}} )}\\ {u_x^{\prime}( {{z_{ - 1}}} )}\\ {{V_z}( {{z_{ - 1}}} )}\\ {V_z^{\prime}( {{z_{ - 1}}} )} \end{array}} \right] &=& {a_{{R_{ - 1}}}}{a_{{R_{ - 2}}}} \cdots {a_{{R_{ - p}}}}\cdots {a_{{R_{ - ( {M - 1} )}}}} \nonumber\\ &&\times\left[ {\begin{array}{@{}*{1}{c}@{}} {{u_x}( {{z_{ - M}}} )}\\ {u_x^{\prime}( {{z_{ - M}}} )}\\ {{V_z}( {{z_{ - M}}} )}\\ {V_z^{\prime}( {{z_{ - M}}} )} \end{array}} \right].\end{eqnarray*}

In the above equations, ${u_x}$ and ${V_z}$ have the same meaning as those in the R-function method $X$ and $V$, and $a_{Rp}^{}$ and $a_{Rp}^{ - 1}$ are the layer matrix and the inverse matrix for the *p*-th layer. Similar to the R-function method, after the coefficients of the air layer are obtained by using the boundary conditions, the values of each component of the surface field can be obtained. Based on the boundary conditions, the Fourier integral of the EM field in each layer can also be derived.

## Supplementary Material

nwab046_Supplemental_File
