# Supplementary material for: Insight into skywave theory and breakthrough applications in resource exploration
Source: Natl Sci Rev. 2021 Mar 23;8(12):nwab046. doi: 10.1093/nsr/nwab046 (PMC8692938; doi:10.1093/nsr/nwab046)
Supplement: nwab046_Supplemental_File [file nwab046_supplemental_file.docx]

# SUPPLEMENTARY DATA

# Insight into skywave theory and breakthrough applications in resource exploration

Qingyun Di1,2,3,4*, Changmin Fu1,2,3,4, Guoqiang Xue3,4,5, Miaoyue Wang^1,2,3,4^, Zhiguo An1,2,3,4, Ruo Wang1,2,3,4, Zhongxing Wang1,2,3,4, Da Lei1,2,3,4, Xianjun Zhuo6

**Corresponding author:* [qydi@mail.iggcas.ac.cn](mailto:qydi@mail.iggcas.ac.cn)

## 1. Establishment of the geoelectrical model at national scale

The national scale model composes of an ionosphere with a resistivity value of 10^4^ Ω·m, an atmosphere with a resistivity value of 10^14^ Ω·m and the height of 100 km, and the subsurface background with a resistivity value of 5000Ω·m. In our following modelling, the east-west aligned transmitting antenna was assumed to be 80 km long with a transmitting current of 200A.

In order to accurately simulate the distribution characteristics of the skywave field in China, we first collected the geological and geophysical data of the entire China region. To balance between the modeling accuracy and computational costs, we selected a grid size of 50 km×50 km in the x- and y-direction. To include the topography in our model, we used a total of 10 grids near the earth surface with a grid height of 500m. We divided the deeper part into 25 grids with a grid height of 1 km. The total number of cells used was 101×80×( 10+25)=286335.

We divided the regional geological structures of the whole country into a number of sections, and simplified the structures for each geological unit to set up the geoelectrical model. We then filled the geological model for the different geological units into the designed mesh and got the whole 3D geoelectrical structures.

## 2. Characteristics of skywave fields generated by a fixed transmitter in China

We designed the models of a half-space, a full space (excluding the displacement current), and a full space (including the displacement current). The numerical results show that the EM field attenuates due to the effect of waveguide. The attenuation of EM field in the model including displacement current is slower, and the propagation distance is longer and the penetration ability is stronger (Supplementary Fig. 1). This not only reveals the propagation mechanism of skywave in the waveguide, but also obtains a new understanding of skywave propagation when considering both the waveguide and the displacement current.


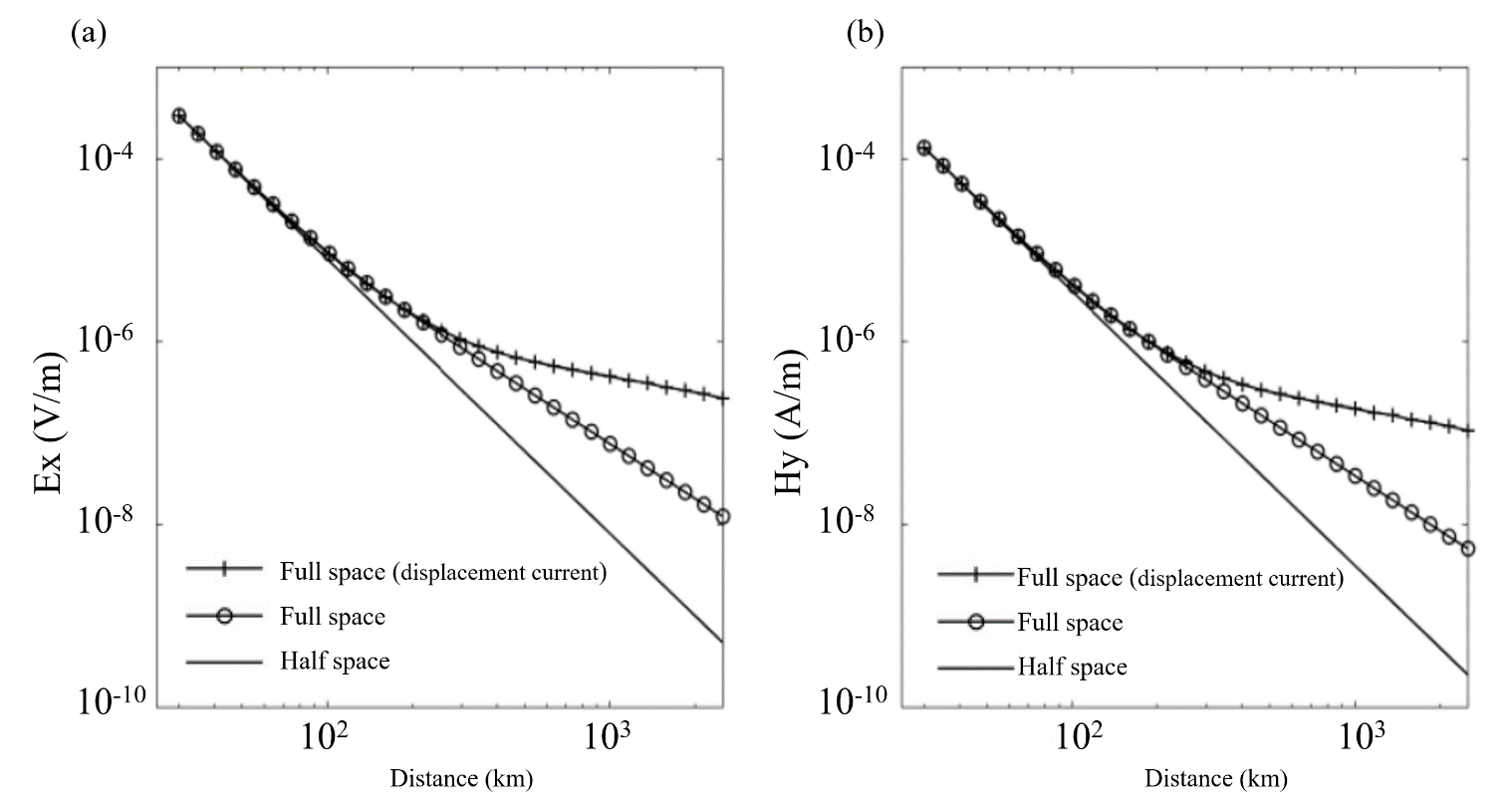


Supplementary Figure. 1 Influence of ionosphere and displacement current on the field strength. (a) Electric field; (b) magnetic field

## 3. Development of skywave data acquisition system

We have developed a multi-channel data acquisition system to perform skywave observations. The system includes a differential amplification with ultra-narrow band filtering to enable extraction of extremely weak EM signal (~0.1 μ V). The system also includes an EM receiver unit with high sensitivity and large dynamic range. The receiver can simultaneously acquire data from 12 input channels. In the end, a total of 30 acquisition systems have been produced.

Supplementary Table 1. Receiver Specification

| **Parameters** | **Performance** |
| --- | --- |
| Channels | 12 channels：3 magnetic and 9 electric channels |
| Frequency range | DC-10kHz |
| Gain | 0.25、1、16、64 |
| A/D resolution | 24 bits |
| Dynamic range | >130dB |
| The input impedance | >10MΩ |
| The input noise | 40nV/$\sqrt{\text{Hz}}$@1Hz |
| The input range | ±10V |
| The processor | ARM+FPGA+DSP |
| Storage capacity | 32GB SD card |
| Synchronization methods | GPS+OCXO |
| Synchronization accuracy | GPS: UTC±25ns；OCXO < ±5×10^-9^ |
| Shell | High strength aluminum alloy shell, waterproof, dustproof |
| Size | 250mm×220mm×125mm |
| Weight | 4.6kg |
| Working temperature | -35℃— +70℃ |
| Power consumption | ≤15W |

We have developed a broadband and low-noise magnetic sensor. The magnetic sensor is the key component of skywave exploration equipment. Since the magnetic sensor can be applied in the military field, this kind of core technology is subject to international blockade. We have tackled core technologies such as high permeability and low loss magnetic core materials, coil winding technology, and weak signal readout circuits, and successfully developed low noise (0.05pT/$\sqrt{\text{Hz}}$@1Hz, international 0.1pT/$\sqrt{\text{Hz}}$@1Hz), broadband (1/10000-10000Hz, international 1/5000-8192Hz) inductive magnetic sensor.

Supplementary Table 2. Magnetic Sensor Specification

| **Parameters** | **Performance** |
| --- | --- |
| Working frequency | 0.0001 Hz–10000Hz |
| The sensitivity | 800 mV/nT, f >> 4 Hz；200 mV/ nT×Hz, f << 4 Hz |
| Noise level | 10 pT/$\sqrt{\text{Hz}}$@ 0.01 Hz；0.05 pT/$\sqrt{\text{Hz}}$@ 1 Hz |
| Size | Length 1226 mm, diameter 75mm |
| Weight | 7kg |
| Working temperature | -30°C ～ +70°C |
| Power consumption | 800 mW |

## 4. Skywave signals in the Biyang depression

Supplementary Fig. 2 shows the signals observed in the Biyang depression. According to the spectrum of field electromagnetic data, the skywave signals are nearly 100 times stronger than the natural field signals.


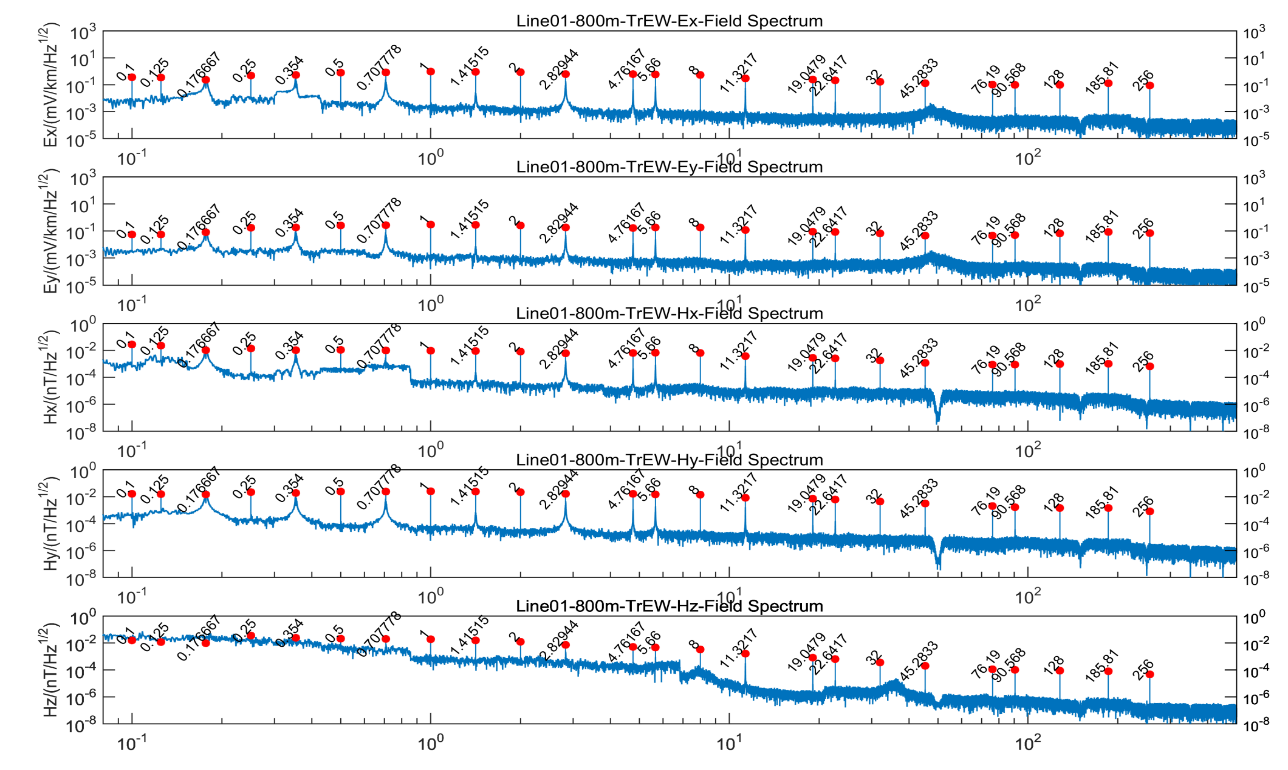


Supplementary Figure. 2 Electrical field Ex and Ey measured in the area of Biyang depression

**5. Comparison of MT, CSAMT and skywave methods**

**5.1 Modelling results of signal propagation**

In order to study the feasibility of the skywave method for mineral exploration, a 1D earth model was computed. The skywave response to the model was compared to the CSAMT and MT responses (Supplementary Fig. 3). At the higher frequencies, all the three methods give similar results as the EM signals travel through the atmosphere from transmitter to receiver. Below approximately 100 Hz, the CSAMT method demonstrates the characteristic near-field effect due to the short distance between the transmitter and the receiver. In contrast, the skywave method show similar plane-wave characteristics to the MT method over the entire frequency range, especially at the low-frequency end. This shows that the skywave method can be used for the detection of deep electrical structures of the earth.

In order to study the data characteristics of the skywave method, we carried out the field experiments in a low-noise area and a high-noise, respectively.


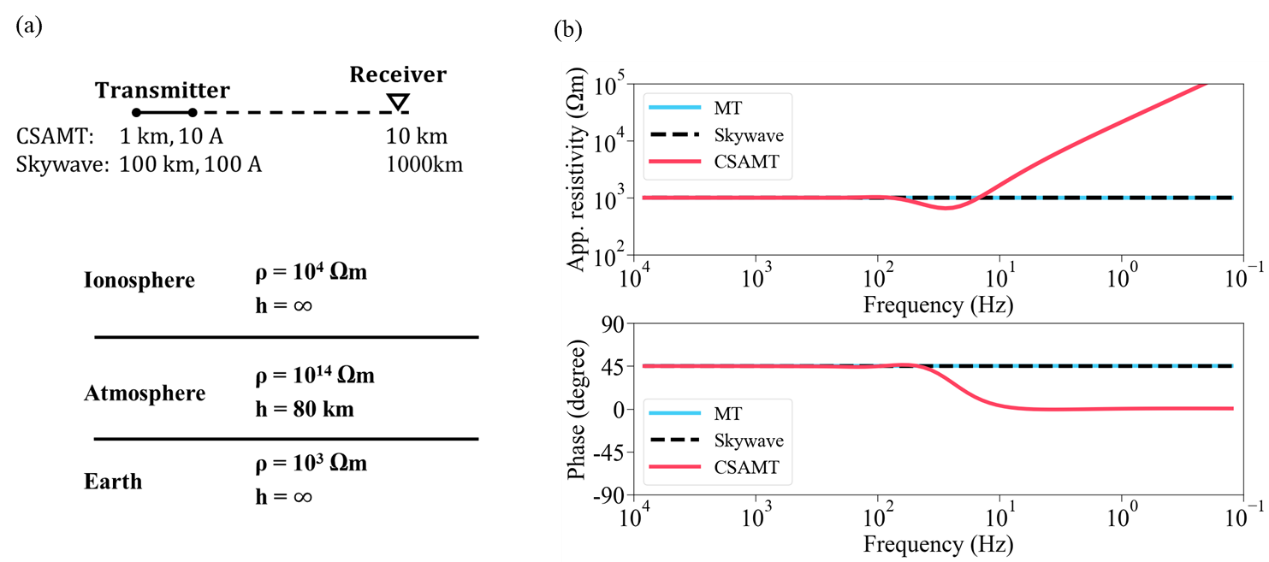


Supplementary Figure. 3 Comparison of forward modelling results between skywave, CSAMT, and MT method. (a)Three layers are assumed in the model: a homogeneous earth half-space with a resistivity of 10^3^ Ω⋅m, an atmosphere with a resistivity of 10^14^ Ω⋅m, and an ionosphere with a resistivity of 10^4^ Ω⋅m. The CSAMT method has a transmitter length of 1 km and a transmitter current of 10 A and a transmitter-receiver offset of 10 km. The source length in the skywave method is 100 km, the transmitting current is 100A, and the transmitter-receiver offset is 1000 km. (b) Simulated sounding curves of skywave, CSAMT and MT method.

**5.2 Field test in a low-noise area**

In the first test, the skywave system was tested in an area with low levels of EM noise, and the acquired data were compared to the MT data using natural EM signals. The sounding curve obtained by the two methods are shown in Supplementary Fig. 4. Because the distances between the survey area and the skywave transmitters were approximately 1200 km, large enough to be considered as a far-field zone, the skywave data should show identical features to the MT data. It can be seen from the figure that the two types of data generally agree well with each other. However, the MT data show outliers at various frequencies, whereas the skywave data are more consistent over the entire frequency range.


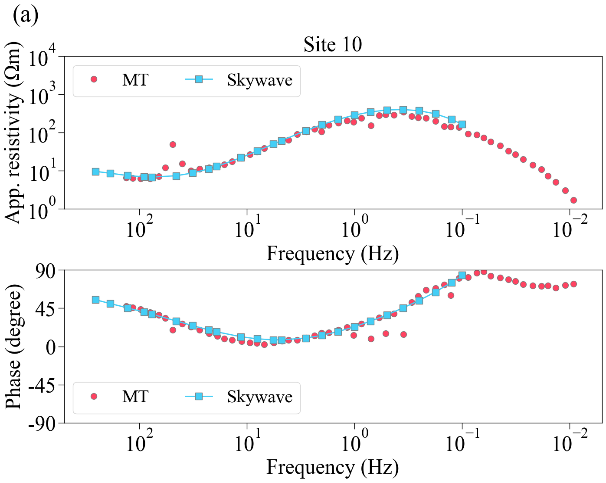

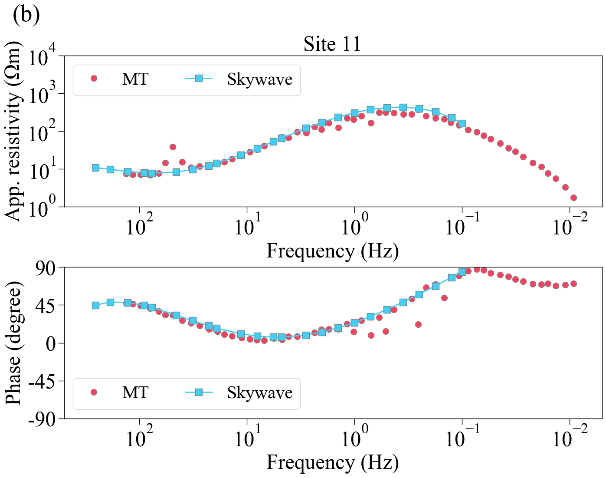

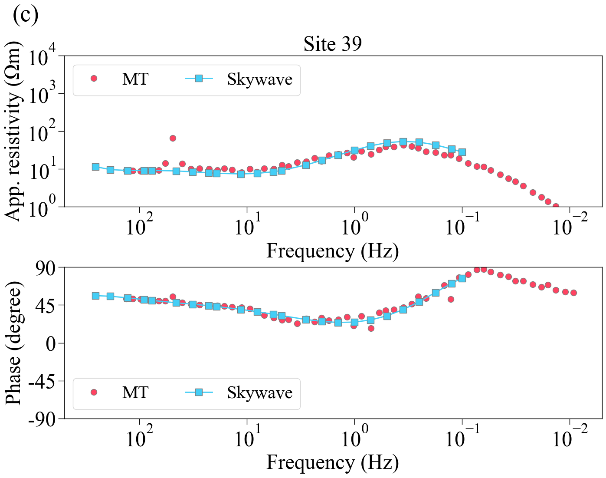

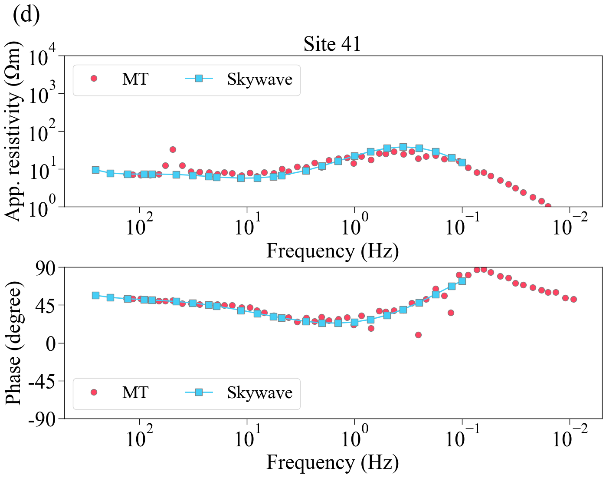


Supplementary Figure. 4 Comparison skywave results with those of MT method in a low-noise area. The sounding curves of sites 10, 11, 39 and 41 are listed as (a), (b), (c), and (d), respectively.

**5.3 Field test in a high-noise area**

Manmade electromagnetic noises can interfere with MT field surveys. The skywave method was investigated in a second, more noisy area. Both the MT and the skywave data are shown in Supplementary Fig. 5. At sounding points 15 and 17, the MT data below 10 Hz (dead-band) are severely affected by the environmental noises. At some sounding points (e.g., 49 and 50), the MT data show the near-field characteristics similar to those of the CSAMT method. In contrast, the skywave data show no evidence of a near field effect, which makes the data more pertinent to the underground structures.


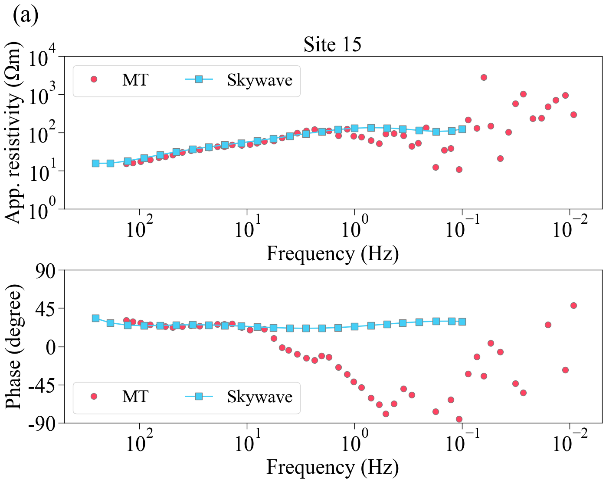

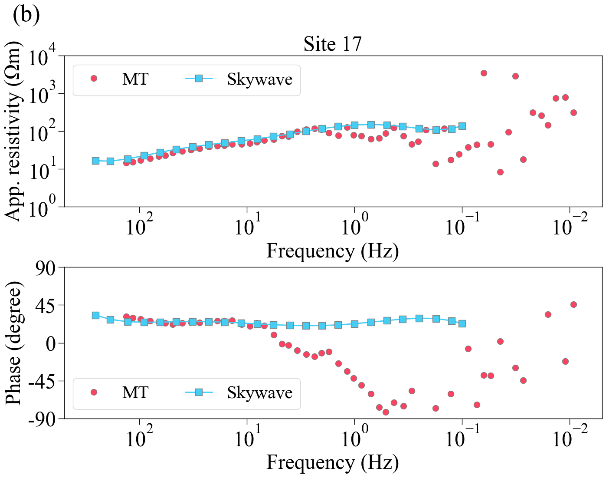

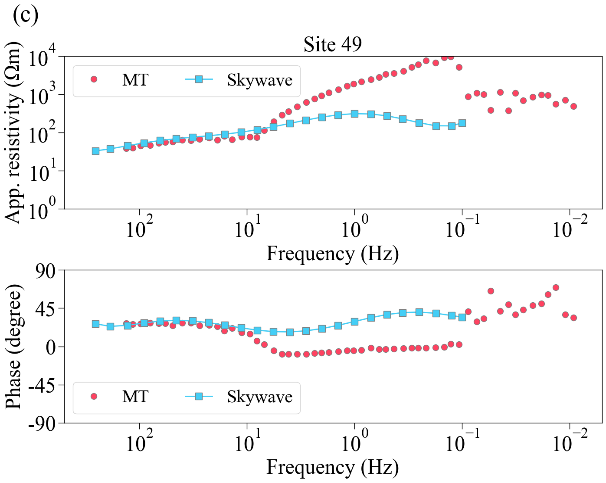

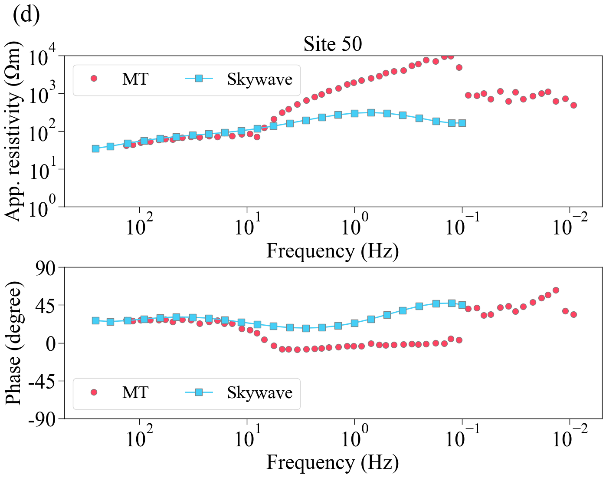


Supplementary Figure. 5 Comparison of the skywave and MT results in a high-noise area. Sounding curves of sites 15, 17, 49 and 50 are listed as (a), (b), (c), and (d), respectively.

In an another example, the skywave method was compared to both the MT and CSAMT methods (Supplementary Fig. 6). It can be seen that the CSAMT method has entered the near-field domain at approximately 32 Hz above which the data no longer reflects the electrical resistivity of the subsurface but is determined by the transmitter geometry. On the other hand, the natural EM signals are weak for the MT method at this frequency. Thus, the MT data have low signal-to-noise ratios, causing the apparent resistivity and phase data fluctuate from frequency to frequency. Furthermore, when the frequency is below 10 Hz, the MT data shows the familiar near-field effect. In contrast, the skywave method produced consistent and quality data in the same are. This example shows that the skywave method can work where neither CSAMT nor MT method may.


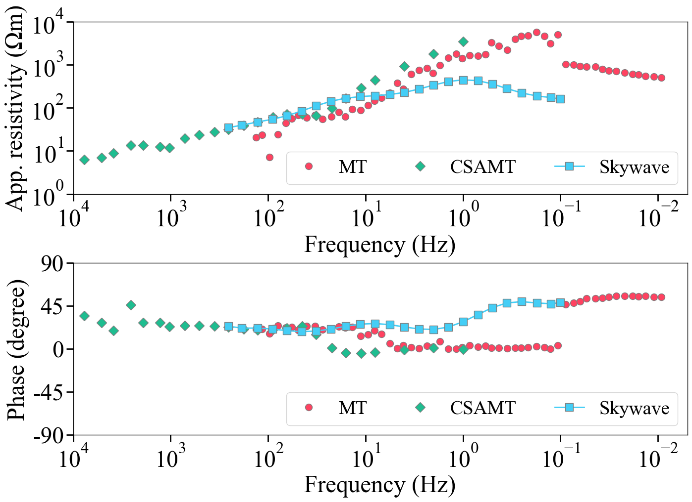


Supplementary Figure. 6 Comparison of apparent resistivity and phase for MT, CSAMT, and skywave exploration. The distance between the transmitter and the receiver is 1160 km.

**6. Transmitting frequencies**

The two frequency tables for the transmitter in our field survey are given in Table 3 and 4.

Supplementary Table 3. Frequency sequence A

| No. | Frequency  (Hz) | No. | Frequency  (Hz) | No. | Frequency  (Hz) | No. | Frequency  (Hz) |
| --- | --- | --- | --- | --- | --- | --- | --- |
| 1 | 309.68 | 13 | 38.095 | 25 | 4.7619 | 37 | 0.59524 |
| 2 | 256.00 | 14 | 32.000 | 26 | 4.0000 | 38 | 0.50000 |
| 3 | 214.93 | 15 | 26.866 | 27 | 3.3582 | 39 | 0.41978 |
| 4 | 185.81 | 16 | 22.642 | 28 | 2.8302 | 40 | 0.35378 |
| 5 | 155.68 | 17 | 19.048 | 29 | 2.3810 | 41 | 0.29762 |
| 6 | 128.00 | 18 | 16.000 | 30 | 2.0000 | 42 | 0.25000 |
| 7 | 107.46 | 19 | 13.433 | 31 | 1.6791 | 43 | 0.20989 |
| 8 | 90.567 | 20 | 11.321 | 32 | 1.4151 | 44 | 0.17689 |
| 9 | 76.190 | 21 | 9.5238 | 33 | 1.1905 | 45 | 0.14881 |
| 10 | 64.000 | 22 | 8.0000 | 34 | 1.0000 | 46 | 0.12500 |
| 11 | 53.731 | 23 | 6.7164 | 35 | 0.83955 | 47 | 0.10494 |
| 12 | 45.283 | 24 | 5.6604 | 36 | 0.70755 | 48 | 0.10000 |

Supplementary Table 4. Frequency sequence B

| No. | Frequency  (Hz) | No. | Frequency  (Hz) | No. | Frequency  (Hz) | No. | Frequency  (Hz) |
| --- | --- | --- | --- | --- | --- | --- | --- |
| 1 | 311.00 | 14 | 39.00 | 27 | 4.10 | 40 | 0.43 |
| 2 | 257.00 | 15 | 33.00 | 28 | 3.46 | 41 | 0.364 |
| 3 | 216.00 | 16 | 27.90 | 29 | 2.93 | 42 | 0.308 |
| 4 | 188.00 | 17 | 23.60 | 30 | 2.48 | 43 | 0.260 |
| 5 | 157.00 | 18 | 20.00 | 31 | 2.10 | 44 | 0.220 |
| 6 | 127.00 | 19 | 17.00 | 32 | 1.78 | 45 | 0.187 |
| 7 | 106.00 | 20 | 14.40 | 33 | 1.52 | 46 | 0.159 |
| 8 | 93.00 | 21 | 12.30 | 34 | 1.29 | 47 | 0.135 |
| 9 | 83.00 | 22 | 10.52 | 35 | 1.10 | 48 | 0.115 |
| 10 | 76.00 | 23 | 8.10 | 36 | 0.85 | 49 | 0.110 |
| 11 | 63.00 | 24 | 6.82 | 37 | 0.72 | 50 | 0.095 |
| 12 | 54.00 | 25 | 5.766 | 38 | 0.61 |  |  |
| 13 | 45.00 | 26 | 4.86 | 39 | 0.51 |  |  |

## 7. Data fits of Biyang depression survey area

Supplementary Figure. 7 shows some typical data fit between the predicted and field raw data. The field data of Biyang survey area is of high quality. The apparent resistivity data were used in the inversion process, and we obtained a final root-mean-square misfit of 3.17 after 21 iterations.


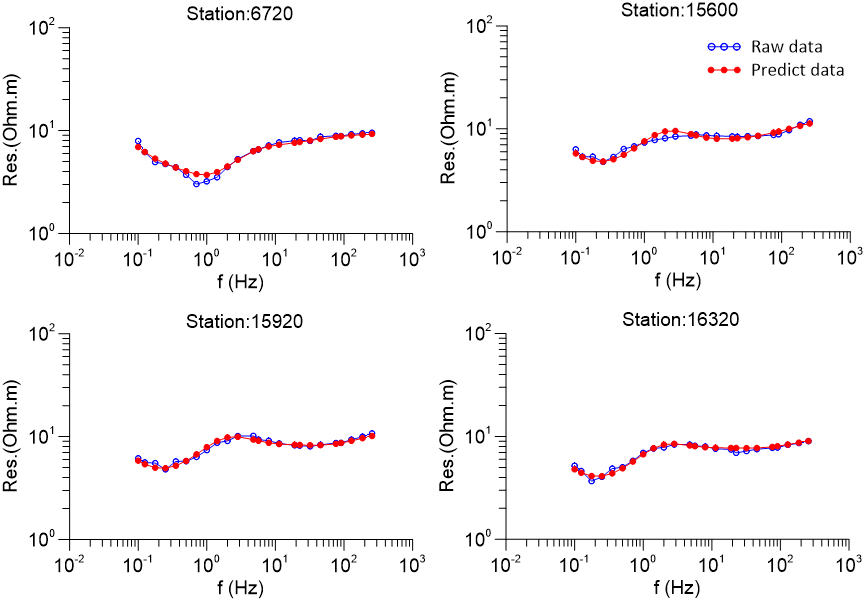


Supplementary Figure. 7. Data fits of the predicted data to the field raw data at different stations
